# Occult deterioration of an aortic annular abscess: how do we diagnose a pseudoaneurysm periaortic valve? A case report

**DOI:** 10.1186/s12872-023-03434-1

**Published:** 2023-08-17

**Authors:** Tienan Chen, Yinling Li, Shuai Qiao, Huaying Fu

**Affiliations:** 1 Department of Cardiovascular Surgery, Second Hospital of Tianjin Medical University, Tianjin, 300211 People’s Republic of China; 2 Tianjin Key Laboratory of Ionic-Molecular Function of Cardiovascular Disease, Department of Cardiology, Tianjin Institute of Cardiology, the Second Hospital of Tianjin Medical University, Tianjin, 300211 People’s Republic of China

**Keywords:** Case report, Infective endocarditis, Aortic annular abscess, Echocardiography

## Abstract

**Background:**

Infectious endocarditis (IE) is a disease caused by the colonization of toxic microorganisms on the endocardium of heart valves [1]. Although much progress has been made in the diagnosis and treatment of IE, its complications, such as annular abscesses [2], still have a high mortality rate. In this case, we describe a patient with infective endocarditis complicated by occult deteriorated aortic annular abscess.

**Case presentation:**

A 44-year-old man was admitted due to weakness of his right limbs and unclear speech for 10 h. He had recurrent fevers for 1 month before admission. Transthoracic echocardiography showed a mix-echoic vegetation attached to the bicuspid aortic valve, moderate aortic regurgitation and a possible aortic annular abscess. Blood cultures were negative and empiric antibiotic therapy was begun. The patient did not have fever again and seem to be clinically improved. However, follow-up transesophageal echocardiography revealed a large periaortic abscess led to aortic sinus pseudoaneurysm. The patient underwent mechanical prosthetic valve replacement and annulus reconstruction successfully. Perivalvular abscess may be insidious deterioration in patients who seem to be clinically improved, which requires us to pay more attention.

**Discussion:**

Occult deterioration of an aortic annular abscess is rare and more attention should be paid. Re-evaluation of echocardiography is required even if the patient’s symptoms improve.

## Background

Infectious endocarditis (IE) is a disease caused by the colonization of toxic microorganisms on the endocardium of heart valves [1]. Although much progress has been made in the diagnosis and treatment of IE, its complications, such as annular abscesses [2], still have a high mortality rate. In this case, we describe a patient with IE complicated by aortic annular abscess.

## Case presentation

**Fig. 1:**
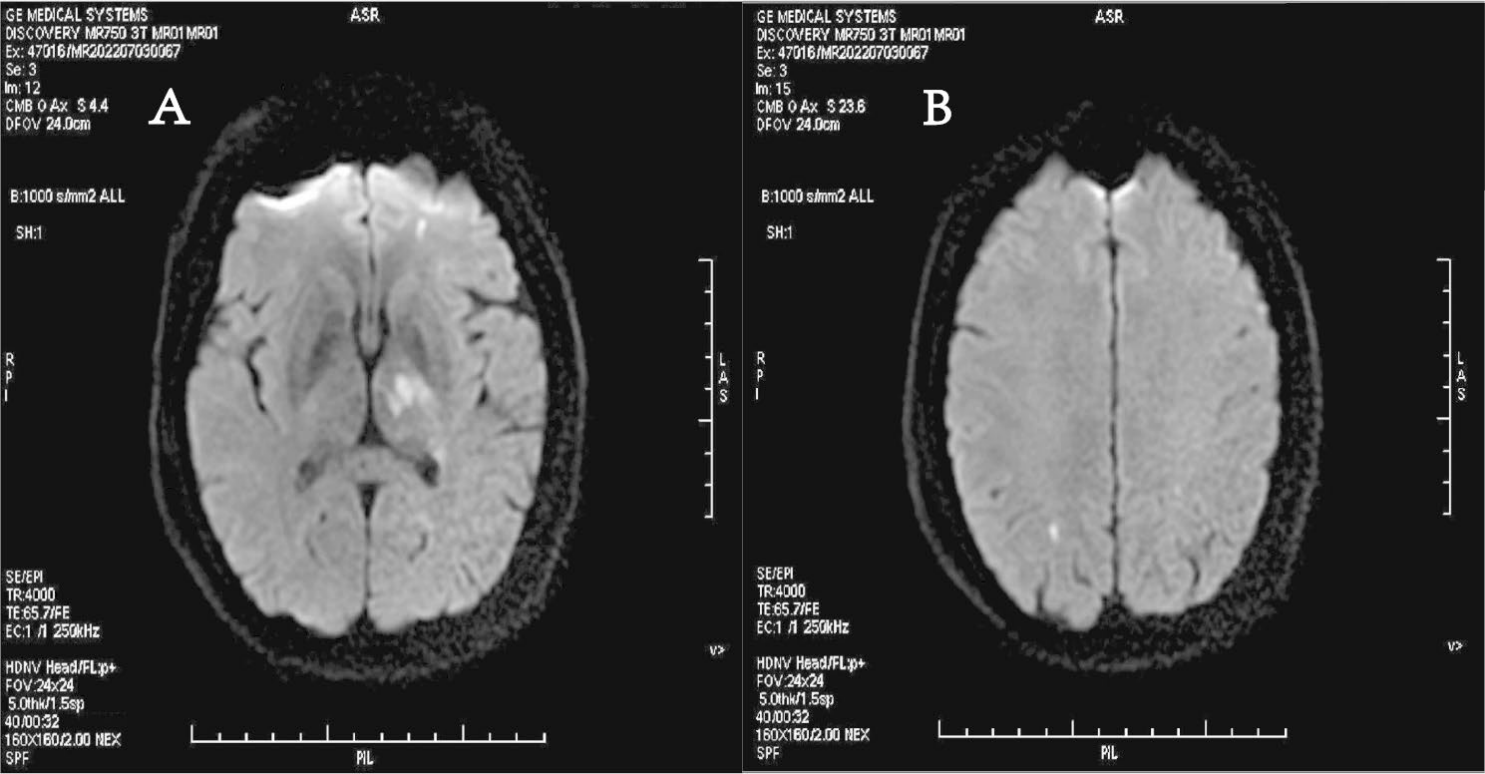
Brain magnetic resonance imaging (MRI) showed patchy and punctate slightly high signals in left basal ganglia and right parietal lobe

A 44-year-old man was admitted presented to the emergency department with a complaint of right limb weakness and unclear speech for the past 10 h. Questioning medical history, he had also repeated fever and was diagnosed diabetes for one month before admission. At the time of physical examination on admission, the patient was delirious, with fever temperature of 38.8 ℃, muscle strength of the right limb was Grade IV, and other signs were stable. Brain magnetic resonance imaging (MRI) revealed left basal ganglia and right parietal lobe cerebral infarction, which is consistent with embolic stroke (Fig. 1).

**Fig. 2:**
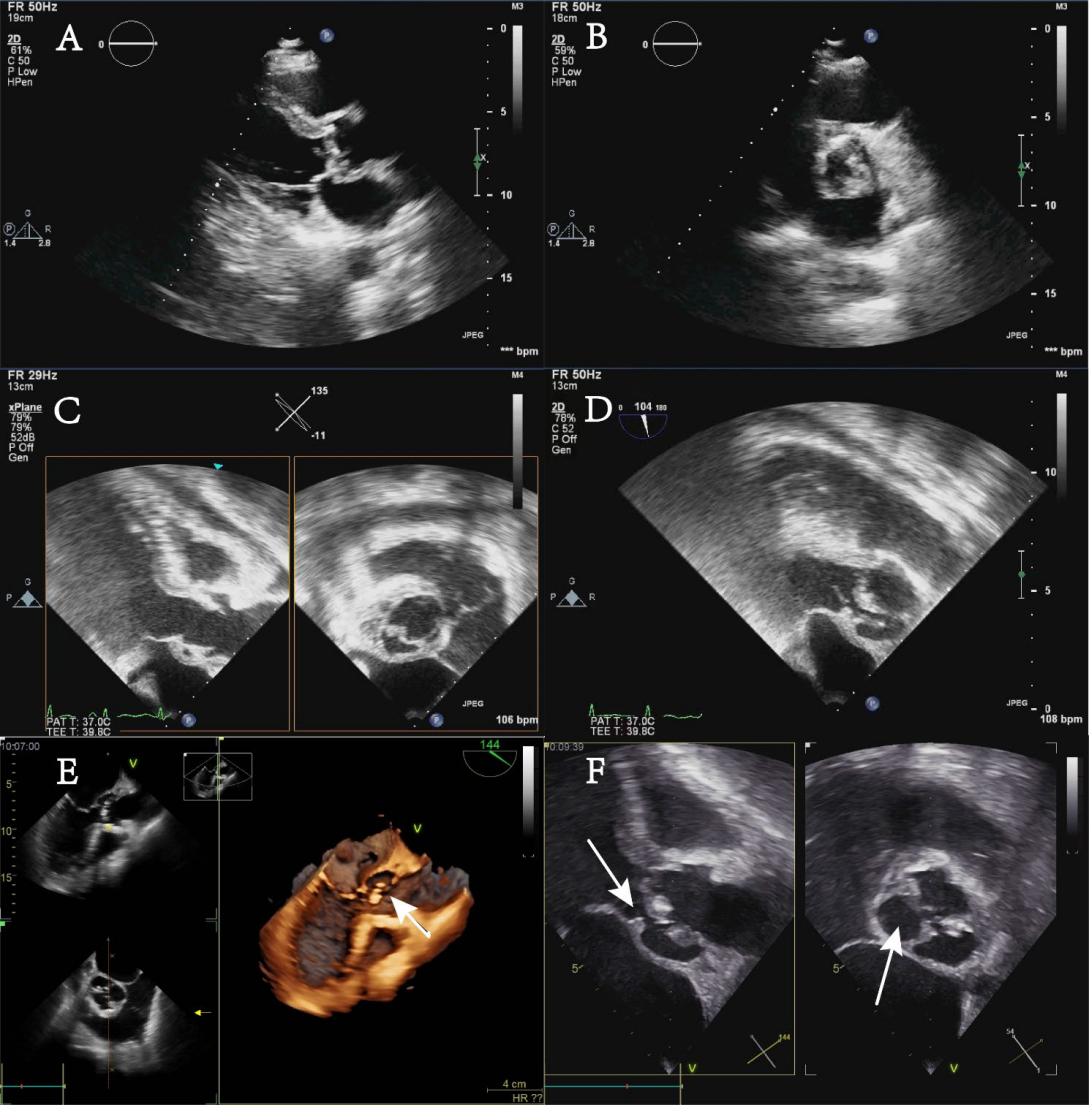
Echocardiographic images at different times. (**A**) Parasternal long axis section of TTE, a 16.4 mm*7.8 mm vegetation can be seen on the right posterior aortic valve. (**B**) Parasternal Aortic Short Axis Right Ventricular Outflow Tract Section of TTE, the aortic valve is calcified bicuspid combined and a possible paravalvular abscess (increased thickening in the right posterior sinus wall of the aorta). (**C** and **D**) The bicuspid aortic valve is arranged in front of and behind, with rough leaflets and multiple mixed echo mass shadows attached to the edge of the leaflet. The maximum echo mass is about 12.4 mm * 6.2 mm in the anterior valve, which is smaller than that seen in TTE 10 days ago. (**E** and **F**) Half a month later since last TEE, a large area of abscess around the aorta leads to a pseudoaneurysm of the sinus of Valsalva, which flows into and out of the left ventricle

Laboratory testing revealed that the blood white cell count was 14.31 × 10^9/L (normal range 3.5–9.5 × 10^9/L), absolute value of neutrophils was 11.16 × 10^9/L (normal range 1.8–6.3 × 10^9/L), percentage of neutrophils was 78.10%. Highly sensitive Troponin I increased to 0.08 ng/ml (normal range 0-0.0268ng/ml) and N-terminal pro-B natriuretic peptide increased to 207.9 ng/L (normal range 0-125ng/L). Chest Computed Tomography (CT) showed that the bilateral lung markings were heavier, the pleura was thickened, strip and nodular high-density shadows were visible, the heart was not enlarged, and calcified spots were visible in the aorta and coronary arteries. Transthoracic echocardiography (TTE) showed the aortic valve was bicuspid combined with calcification, moderate regurgitation and a possible paravalvular abscess (increased thickening to 5 mm in the right posterior sinus wall of the aorta) (Fig. 2A and B). A 16.4 mm*7.8 mm vegetation can be seen on the right posterior aortic valve. Based on the clinical, laboratory, and echocardiographic findings, the patient was diagnosed infective endocarditis (IE). According to the AHA/ACC guideline for the management of patients with valvular heart disease, delaying valve surgery for at least 4 weeks may be considered for patients with IE and major ischemic stroke if the patient is hemodynamically stable [3], the patient was prescribed antibiotics with vancomycin 0.5 g per every 6 h. The transesophageal echocardiography (TEE) was performed 10 days later revealed that the vegetation was smaller than before (Fig. 2C and D). He had no fever again and no complaints of discomfort and was hemodynamically stable, the blood cultures were negative twice.

However, TEE one month later after cerebral infarction revealed a large periaortic abscess (Fig. 2E F, arrow) led to formation of an aortic sinus pseudoaneurysm and flows were in from pseudoaneurysm and out to left ventricular combined with moderate mitral valve regurgitation.

**Fig. 3:**
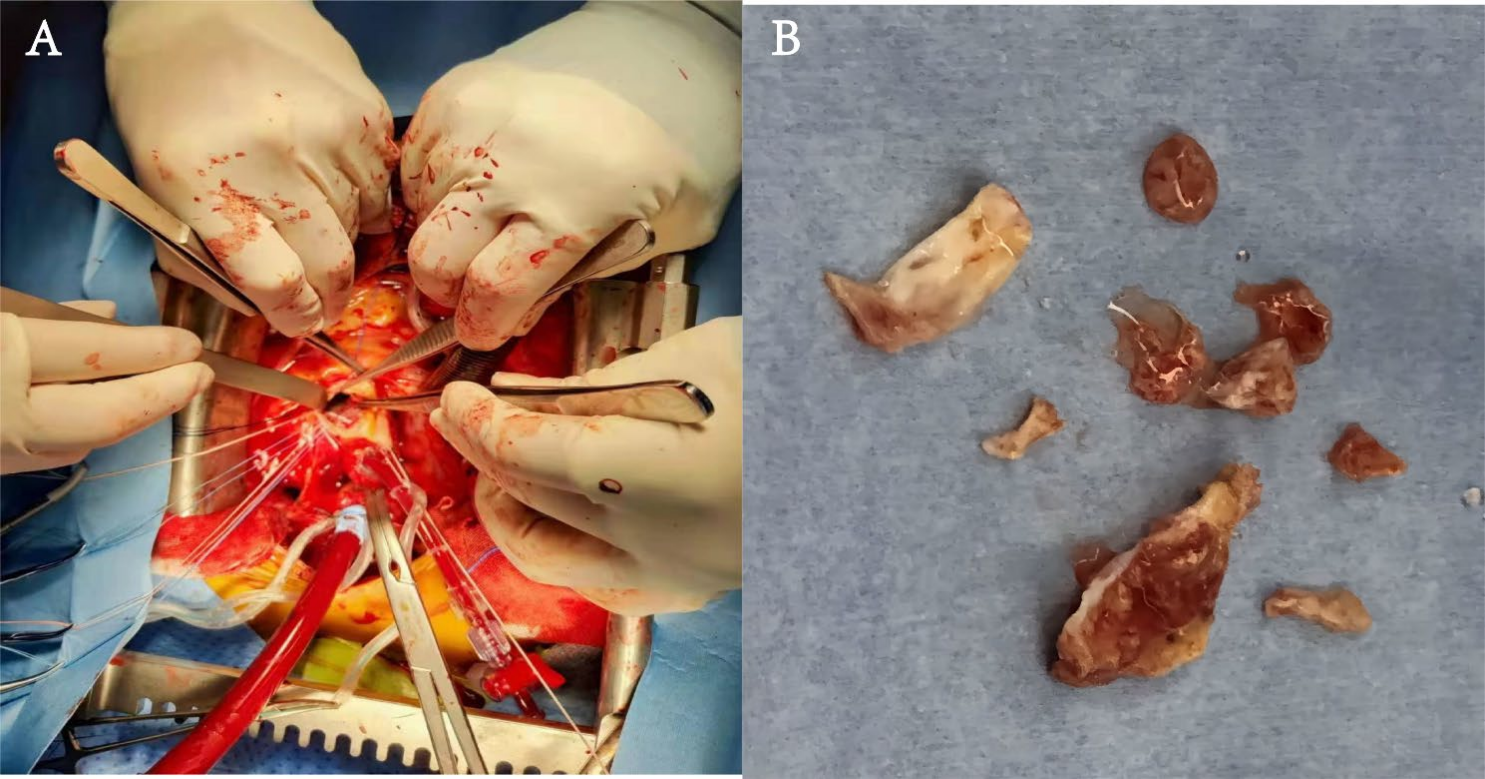
During the operation, (**A**) Bicuspid deformity combined with vegetation. Aortic annular abscess eroded to the bottom of the anterior lobe of the mitral valve, causing prolapse of the mitral valve annulus. (**B**) Aortic valve vegetations and perivalvular abscesses were completely removed

Cardiopulmonary bypass and aortic valve replacement surgery via median sternotomy were performed. During the operation, it was confirmed that bicuspid deformity combined with vegetation, an aortic annular abscess eroding into the base of the anterior mitral leaflet making prolapse of mitral valve annulus (Fig. 3). Aortic valve vegetations and perivalvular abscesses were completely removed. 5/0 Prolene suture was used to continuously suture bovine pericardium to reconstruct mitral aortic valve fiber connection and fix the anterior mitral valve annulus. The patient underwent mechanical prosthetic valve replacement and annulus reconstruction successfully. The tissue culture of the diseased aortic valve showed no bacterial growth and no pathogenic microorganism was identified. The patient’s condition was stable after operation. He was discharged two weeks later with antibiotics for six-weeks. During the one-month follow-up, the patient felt well, laboratory testing revealed that the blood white cell count and percentage were normal.

## Discussion

In the present case, the patient was admitted due to acute cerebral infarction, which was considered to be caused by vascular embolism due to the shedding of vegetations from infective endocarditis. Embolization event is one of the common life-threatening serious complications in patients with infective endocarditis, which is caused by vegetation falling off from the moving valve, with an incidence of 13 − 49% [4].

A lot of patients have developed an aortic pseudoaneurysm after an abscess developed. This condition is caused by the drainage and cavitation into the aorta [5]. The formation of periaortic abscess is a dynamic process [6] that can lead to the formation of a pseudoaneurysm or a vasculature. It can start with the aortic wall being thickened [5]. When antibiotics are used before sampling or endocarditis is caused by pathogens with slow growth or demanding culture medium, it can lead to negative blood culture [7]. On the other hand, Coxiella burnetii is a common cause of blood culture–negative IE [8]. It was confirmed a higher mortality in patients with culture-negative IE (CNIE) compared with those culture-positive (CPIE), 30-day mortality was approximately 5% higher in CNIE than CPIE patients [9]. The development of aortic annular abscess caused by infective endocarditis in the present patient is occult and rare. Perivalvular abscess may be insidious deterioration in patients who seem to be clinically improved. The early manifestations of infective endocarditis are lack of specificity. The development of aortic annular abscess caused by infective endocarditis in the present patient is occult and rare. Perivalvular abscess may be insidious deterioration in patients who seem to be clinically improved, which required us to pay more attention. We have experienced the evolution of the patient, which made it easier for us to deduce the diagnosis of the patient. If the patient’s third echocardiogram is the first echocardiogram, how should we diagnose? Infective endocarditis should be considered when pseudoaneurysm or abnormal tunnel near aortic valve were found.

## Conclusion

To sum up, patients with culture-negative infective endocarditis had higher mortality than those with culture-positive [9]. They may have serious complications even if the vegetations minimized after the antibiotic’s treatment. Infective endocarditis should be considered when pseudoaneurysm or abnormal tunnel near aortic valve were found.

### Learning objectives

1. Perivalvular abscess may be insidious deterioration in patients who seem to be clinically improved, which requires us to pay more attention.
2. Patients with culture-negative infective endocarditis may have serious complications even if the vegetations minimized after the antibiotic’s treatment or the patient is hemodynamically stable.
3. Infective endocarditis should be considered when pseudoaneurysm or abnormal tunnel near aortic valve were found.

## Data Availability

All data generated or analysed during this study are included in this published article.

## Abbreviations

IE: Infectious endocarditis
MRI: magnetic resonance imaging
CT: Computed Tomography
TTE: Transthoracic echocardiography
TEE: transesophageal echocardiography
CNIE: culture-negative infectious endocarditis
CPIE: culture-positive infectious endocarditis
